# Effectiveness of Dance Movement Therapy in the Treatment of Adults With Depression: A Systematic Review With Meta-Analyses

**DOI:** 10.3389/fpsyg.2019.00936

**Published:** 2019-05-03

**Authors:** Vicky Karkou, Supritha Aithal, Ania Zubala, Bonnie Meekums

**Affiliations:** ^1^Faculty of Health and Social Care, Faculty of Arts and Sciences, Edge Hill University, Ormskirk, United Kingdom; ^2^Department of Performing Arts, Faculty of Arts and Sciences, Edge Hill University, Ormskirk, United Kingdom; ^3^Division of Rural Health and Wellbeing, Institute for Health Research and Innovation, University of the Highlands and Islands, Inverness, United Kingdom; ^4^Independent researcher, Manchester, United Kingdom

**Keywords:** dance movement therapy, depression, effectiveness, systematic review, meta-analysis

## Abstract

**Background:** Depression is the largest cause of mental ill health worldwide. Although interventions such as Dance Movement Therapy (DMT) may offer interesting and acceptable treatment options, current clinical guidelines do not include these interventions in their recommendations mainly because of what is perceived as insufficient research evidence. The 2015 Cochrane review on DMT for depression includes only three studies leading to inconclusive results. In a small and underfunded field such as DMT, expensive multi-centered Randomized Controlled Trials (RCTs) are as yet rare. It is therefore, necessary to not only capture evidence from RCTs, but to also look beyond such designs in order to identify and assess the range of current evidence.

**Methods:** We therefore conducted a systematic review of studies that aimed to explore the effectiveness in the use of DMT with people with depression. This led to a qualitative narrative synthesis. We also performed meta-analyses that calculated the effect size for all included studies, studies with RCT designs only, followed by a subgroup analysis and a sensitivity analysis. In all meta-analyses a random effects model was used with Standardized Mean Differences (SMD) to accommodate for the heterogeneity of studies and outcome measures.

**Results:** From the 817 studies reviewed, eight studies were identified as meeting our inclusion criteria. Three hundred and fifty one people with depression (mild to severe) participated, 192 of whom attended DMT groups while receiving treatment as usual (TAU) and 159 received TAU only. Qualitative findings suggest there was a decrease in depression scores in favor of DMT groups in all studies. Subgroup analysis performed on depression scores before and 3 months after the completion of DMT groups suggested changes in favor of the DMT groups. When sensitivity analysis was performed, RCTs at high risk of bias were excluded, leaving only studies with adult clients up to the age of 65. In these studies, the highest effect size was found favoring DMT plus TAU for adults with depression, when compared to TAU only.

**Conclusions:** Based on studies with moderate to high quality, we concluded that DMT is an effective intervention in the treatment of adults with depression. Furthermore, by drawing on a wide range of designs with diverse quality, we were able to compile a comprehensive picture of relevant trends relating to the use of DMT in the treatment of depression. Despite the fact that there remains a paucity of high-quality studies, the results have relevance to both policy-making and clinical practice, and become a platform for further research.

## Background

### Rationale: Why Is It Important to Do This Review

According to the World Federation for Mental Health (WFMH, 2012), depression is the largest cause of mental ill health worldwide, described as a “global burden” (Scott and Dickey, 2003) or a “global crisis” (WFMH, 2012). Similarly, the World Health Organization (WHO, 2017) indicated that more than 350 million people of all ages are faced with depression as a clinical diagnosis. This condition differs from just feeling “low” or experiencing mood swings in response to daily life events; serious depression can affect people in multiple ways and can be disabling to the individual and disruptive to family and whole communities. According to the American Psychiatric Association (APA, 2000), for a diagnosis of major depression, five or more of the following symptoms are needed in the same 2 week period, causing significant distress or impairment of functioning: low mood, loss of interest or pleasure in most activities, sleep disturbances, changes in appetite or unintentional changes of weight, decreased energy, either slowed or agitated movement, decreased concentration and in some cases, feelings of guilt, worthlessness and thoughts of suicide.

In England and Wales, the current draft guideline from the National Institute for Health and Care Excellence (NICE, 2018) for adults suggests that talking therapies and medication are the most effective treatment options. The Scottish Intercollegiate Guidelines Network (SIGN, 2010), the Scottish equivalent to NICE, makes similar suggestions. Amongst the psychological treatment options for depression recommended by the new National Institute for Health and Care Excellence (NICE, 2018) draft guideline, cognitive behavioral therapy, interpersonal psychodynamic psychotherapy, counseling for depression, short-term psychodynamic therapy and couples therapy are mentioned. Exercise has also made its way into the guidelines for less severe depression.

However, despite the prevalence of multiple and, often, body-based symptoms in depression, non-verbal and creative types of psychotherapy such as Dance Movement Therapy (DMT)^1^ are not among the recommended treatment options. It is possible that this is because to date systematic reviews of research studies including Meekums et al. (2015) have not been able to draw confident conclusions of the effectiveness of this intervention for clients with depression; the low number and heterogeneity of studies available have been reported as reasons for inconclusive results. Reviews of research that allow for confident conclusions are increasingly required by policy makers in order to justify resource allocation. However, the question of whether limited research evidence should be taken to indicate limited effectiveness is highly debatable. Altman and Bland as early as (1995) argued that “When we are told that ‘there is no evidence that A causes B' we should first ask whether absence of evidence means simply that there is no information at all.” (p. 485). The same authors also suggest that when there is data, even non-significant results need to be considered for their clinical significance, especially for new treatment options.

This systematic review is, therefore, not just important for facilitating efficient integration of information into policy making in adult services; it is also necessary to demonstrate clearly and transparently where the effects of DMT are consistent and how they vary across contexts in order to translate research findings to clinical practice. Meta-analysis can provide more precise estimates than individual studies, minimizing bias and reducing chance effects. DMT is a relatively new intervention with an emerging evidence base. It is necessary to evaluate the wider range of available evidence stemming from different types of study designs alongside emerging new data, to allow for decision-making that is based on the totality of available evidence, whilst checking for consistency of results across designs.

### A Critique of What Constitutes “Evidence”

The definition of what constitutes good evidence is debated at length, especially for psychotherapy. Randomized Controlled Trial (RCT) is the design that is generally perceived as the golden standard for establishing effectiveness (Higgins et al., 2017). It has however been questioned if this is the only and an appropriately fitted design for research in psychotherapy (Clay, 2010; Holttum and Huet, 2014). For example, in RCTs there is often an expectation that intervention groups will consist of participants with one set of diagnoses, who are randomly allocated to certain groups, a premise that clashes with regular psychotherapy practice. Participants with mixed diagnosis and other co-morbid characteristics are common amongst those receiving group psychotherapy. The overall group fit is an important concern in regular group psychotherapy. In contrast, pre-stated single-diagnosis inclusion criteria and randomization in RCTs tend to ignore these common group practices.

Furthermore, there have been arguments that studies in the field are not sufficiently powered to detect true differences (Leichsenring et al., 2017). Calculating changes within groups, i.e., before and after therapy, may have limitations (Eysenck, 1963; Cuijpers et al., 2016), but may also accommodate for the smaller power of the studies in the field.

According to Shean (2014), RCT design favors treatment options that are simple and deal with uncomplicated symptoms. In contrast, most psychotherapists argue that they offer complex interventions to clients with complex needs. In the UK, the Medical Research Council (MRC) (Craig et al., 2008) acknowledges that complex interventions present additional challenges when designing studies of effectiveness. They suggest that there are several phases in evaluating such interventions which do not need to be followed linearly. Although experimental and RCT designs are highly valued, practical applicability needs to be considered. In all cases, demonstration of an understanding of the process is important, as evidenced by a clear description of the intervention. The MRC report, while highlighting the importance of a focus on outcomes and attempts at standardization, recommends the adaptation of the study to local circumstances and context.

Without diminishing the importance of RCTs, the value of looking at a broad range of evidence and alternative research designs when it comes to policy making and evidence-based practice has been argued extensively (Shadish et al., 2001, 2008; Kazdin, 2010). Some researchers suggest that quasi experiments may provide useful information about the potential effectiveness of an intervention (Colliver et al., 2008). Either way, the advantage of randomization is that it can prevent selection bias and reduce the difference between groups on both known and unknown confounding variables. Even without randomization, studies can still reflect many other aspects of therapy.

In all cases systematic reviews remain important and highly valued summaries of evidence of effectiveness, the most respected being reviews published by the Cochrane Collaboration (Higgins et al., 2017).

### Evidence in the Treatment of Depression

In the treatment of depression, existing evidence from Cochrane reviews covers both the effectiveness of anti-depressant medication, different types of talking therapies and cognitive behavioral therapy in particular. While there are systematic reviews that provide evidence for the value of these interventions (Arroll et al., 2009; Hetrick et al., 2012; Rummel-Kluge et al., 2015; Davies et al., 2018), there are some that present a critical perspective on these prevalent approaches. For example, Arroll et al. (2009), in their review of the use of anti-depressant medication, acknowledge that the side effects of medication are not sufficiently reported. Others, such as Shinohara et al. (2013), report that the benefits and harms of behavioral therapy are not appropriately shared raising concerns around participant responses and withdrawal. Reviews on other forms of psychotherapy, for example from Abbass et al. (2014), highlight the value of psychodynamic interventions, arguing that there are sustained benefits after 3 months and after 6 months. Furthermore, there is a growing body of research literature that provides evidence for the value of different psychotherapy and counseling approaches when compared to cognitive behavioral therapy including short term psychodynamic psychotherapy, generic counseling and counseling for depression (Ward et al., 2000; Richards and Bower, 2011; Cuijpers et al., 2013; King et al., 2014; Freire et al., 2015; Pybis et al., 2017; Steinert et al., 2017).

Differences between types of client populations affected by depression have also been reported in the literature. For example, Dennis and Hodnett (2007) found that psychological and psychosocial interventions were more effective than usual care for women with postnatal depression. With children and adolescents, Cox et al. (2014) found it more difficult to establish a clear superiority of psychological interventions over antidepressant medication. They did however, raise high risk of suicidal thoughts in association with antidepressant medication, making psychological interventions potentially safer interventions to use. For older people with depression, the review by Wilson et al. (2008) concluded that cognitive behavioral and psychodynamic therapies were comparable and both potentially useful.

However, these approaches rely heavily on verbal interaction. Exercise, although not a form of psychotherapy, offers a non-verbal approach to the treatment of depression that is gaining popularity, finding its way into the 2018 draft guideline from NICE. Still, the Cochrane review of the literature on this topic by Cooney et al. (2013) suggests that the effect was small and did not seem to have long lasting effects. The same review also reported that attendance rates ranged from 50 to 100%, indicating the possibility of high attrition rates.

Given that available treatments may not be the treatment of choice for certain clients and/or client populations and there might be concerns about adverse effects as is the case with the use of medication, there is an urgent need to explore the evidence from diverse treatment options. DMT is one such option.

### Dance Movement Therapy (DMT): Description of the Intervention

In the UK, DMT receives regulation via the UK Council of Psychotherapy (UKCP), one of the main regulatory bodies of psychotherapists. However, unlike verbal psychotherapy, and unlike the most prevalent forms of psychotherapy recommended for depression such as cognitive behavioral therapy, DMT does not require considerable cognitive and linguistic skills from the client/patient. Therefore, it can potentially bypass social or cultural barriers. Karkou and Sanderson (2006) argue that DMT, alongside other arts therapies (art therapy, drama therapy, and music therapy are the other arts therapies practiced in the UK) offers an attractive option for clients since it allows them to work through issues that are located at a non- and pre-verbal level. Thus, DMT may offer a way to work through issues that are difficult to articulate or are buried in the unconscious because they are painful, frightening, or simply difficult to access and address through cognitive means.

DMT as a form of psychotherapy is extensively discussed by authors such as Meekums (2002), Karkou and Sanderson (2006), Payne (1992), Payne (2006), and Levy (1988). In particular, Meekums (2002) discusses DMT as a *creative* form of psychotherapy. Following on from her theory-generating doctoral research (Meekums, 1998), Meekums (2002) argues that the therapeutic process follows the same pathway as the creative process. This process comprises the following phases: preparation, incubation, illumination, and evaluation. Moreover, she identifies the central importance of the movement metaphor within this process, including its links to body memory, body language, and mediation of the therapeutic relationship.

DMT has also been researched as one of the arts therapies by Karkou and Sanderson (2006) for example, who reported on survey results (Karkou, 1998) that explored similarities and differences between DMT and the other arts therapies. This study argued, amongst other things, that DMT shares with the other arts therapies similar overall therapeutic approaches, namely humanistic, psychodynamic, developmental, artistic/creative, active/directive, and eclectic/integrative therapeutic approaches. An updated survey 17 years later (Zubala, 2013; Zubala et al., 2013; Zubala and Karkou, 2015) suggests that these trends remain largely unchanged. However, similar to the work by Meekums (1998, 2002), these studies focus on defining the field and identifying relevant processes and do not attempt to answer questions of effectiveness.

With regards to effectiveness, Cochrane reviews in DMT with different client groups are available, albeit often with a small number of studies included. For example, next to the Cochrane review on depression mentioned above (Meekums et al., 2015), there are Cochrane reviews on DMT for schizophrenia (Ren and Xia, 2013), cancer care (Bradt et al., 2015), and dementia (Karkou and Meekums, 2017), none of which had more than three studies included due to the strict inclusion criteria posed by the Cochrane Collaboration. The difficulty in capturing the effectiveness of this field with different client populations when the included studies were limited to designs of RCTs is apparent in these highly stringent systematic reviews.

In contrast, a larger number of studies was included in the meta-analysis by Koch et al. (2014) not confined to RCTs. Of the total 23 studies included, ten studies with RCT and controlled trials included measures of depression (total scores or subscales). A moderate effect of DMT and dance on depression was reported. However, in addition to the diverse research designs, populations were equally diverse (not confined to depression), and interventions included any form of dance practice (not solely DMT).

### DMT and Depression: How the Intervention Might Work

Following an early scoping review of the literature (Mala et al., 2012) that identified a number of empirical research studies on the effectiveness of DMT for depression, the Cochrane review on this topic was completed (Meekums et al., 2015). The Cochrane review identified three studies that met the criteria for inclusion (147 participants). A sub-group analysis suggested that for adults, there was evidence of a positive effect for DMT in reducing depression. However, the evidence was too thin to allow any firm conclusions due to the low number of studies (and associated number of participants) and the varying, generally low, quality.

Another important contribution of this Cochrane review was that it hypothesized on the reasons of why this intervention could be useful for depression and identified several “active ingredients” as follows:

#### Participating in Dance as an Art Form and as Exercise

The authors discussed the potential contribution of dance as a central component of DMT to generate vitality, even joy, for clients who, due to their depression, lacked animation. They supported this claim with reference to a seminal theoretical article by Schmais (1985) and recent empirical studies including Koch et al. (2007). Further arguments can be made regarding dance participation due to physiological responses associated with exercise such as the excretion of endorphins, the enhancement of chemical neurotransmitters (Jola and Calmeiro, 2017) and the active engagement of almost every part of the brain (Bläsing, 2017).

The positive contribution of music and music therapy in decreasing levels of depression has already been demonstrated (Aalbers et al., 2017). Although music is not an essential component of dance practice in a DMT context, its regular use may act as a supporting component to the central active ingredient of dance with this client population.

#### Building the Therapeutic Relationship/s Through Mirroring

The presence of a therapeutic relationship is a key difference between dance as a sensitive form of teaching or community practice on the one hand and DMT as a form of psychotherapy on the other (Karkou and Sanderson, 2000, 2001, 2006; Meekums, 2002). This relationship is also highly valued as an agent of change for clients with depression who often experience isolation and loneliness. Literature in humanistic and existential approaches to psychotherapy (e.g., Yalom, 1980; Rogers, 1995) suggests that a meaningful interaction is central to the therapeutic process. In more recent years several psychotherapists argue that this relationship is also the main agent of therapeutic change and directly linked with therapeutic outcomes (Ardito and Rabellino, 2011; Stamoulos et al., 2016).

In DMT, the therapeutic connection can take the shape of an embodied relationship, particularly present in the model developed by Chace (Chaiklin and Schmais, 1986). The technique of mirroring^2^ is frequently used in this practice as a way of enhancing embodied relationships (McGarry and Russo, 2011; Fischman, 2015) and discussed with regards to studies in neuroscience (Meekums, 2002; Berrol, 2006; Rova, 2017). The sensori-motor mirroring system (Rizzolatti et al., 1996; Gazzola et al., 2006) for example, appears to be particularly relevant, offering an additional explanation of the mechanism behind the technique of mirroring, though neuroscience does not fully explain the psychological processes behind this complex practice.

#### Accessing Unconscious Material Through Imagination, Symbolism, and Metaphor

Another reason why DMT might be an effective intervention is its capacity to tap into unconscious, hard to reach or taboo feelings and thoughts. Imagery, symbolism and metaphors are important DMT tools in this process. Activating imagination is a component of DMT that was discussed as early as Dosamantes-Alperson (1981) in the context of the approach known as Authentic Movement^3^ (Whitehouse, 1979; Pallaro, 2007; Chodorow, 2013). Active imagination allows access to difficult feelings, and anger in particular, which for people with depression may be internalized, attacking one's own self (Freud, 1917). With this psychodynamic explanation of depression in mind, it is therefore possible that imagination might act as a vehicle to express difficult emotions and, through symbolism and metaphor, to process them in a safe way, finding resolutions to one's underlying difficulties (Meekums, 2002; Karkou and Sanderson, 2006). This proposition suggests that DMT could have profound and long-lasting effects that are not present for interventions that do not address the underlying reasons for depression.

#### Achieving Integration Through Reflection, Creativity, and Movement Narratives

As early as 1985, Schmais argued that integration between mind and body is a key therapeutic factor for DMT. Integration is still a term used in the ADMP UK (2013) definition of the discipline as the overall aim of the work. In practice integration can happen through reflecting on movement material that may or may not be congruent with one's own thoughts and feelings. Exploring new and unexpected connections between known things, i.e., engaging in a creative process (Karkou and Sanderson, 2006), can also have an integrative character. Finally, summarizing one's experience of therapy in a movement sequence or a symbolic posture or gesture can act as an essential and potent reference back to the process of therapy. Movement material can therefore, act as a form of story-telling, a movement and embodied narrative of key moments in the therapeutic journey (Karkou, 2015).

The formation of links between body, thoughts and feelings becomes important for people with depression who may experience a disconnect between what they feel, think and/or do. Integration, an important outcome for a number of different forms of psychotherapy including integrative psychotherapists such as Norcross and Goldfried (2005), may therefore, be another important “active ingredient,” which can be relevant to and responsible for therapeutic change.

### Researching DMT Practice

These “active ingredients” presented above appear to respond and add to therapists' views on the topic as explored by the survey of practitioners by Zubala et al. (2013) and clients' experiences of the DMT process (Genetti, 2011). While there is growing research evidence in the field (Zubala and Karkou, 2018), DMT remains largely under-funded and thus, under-researched. RCTs, the gold standard for assessing the effectiveness of an intervention (Higgins et al., 2017), require resources that are often beyond the reach of many researchers in the field, including those associated with time, money, access to large numbers of clients, specialist clinical trials support, and control over the environment in which a study can take place. Furthermore, as discussed above, RCTs pose limitations when applied to complex interventions. Since DMT is indeed a complex intervention, other research approaches need to also be considered. Quasi-experimentation, a predominant approach adopted in DMT research, may reveal important information that is typically overlooked and omitted from many systematic reviews. In this review we attempt to change this.

### Overall Aim

To explore evidence of effectiveness in the use of DMT with people with a diagnosis of depression.

### Research Questions

The main research question we asked for this study was:

- Is DMT effective for clients with a diagnosis of depression?

We were also interested in the following sub-questions:

1. What patterns emerge from the collected evidence relating to the severity of depression, the setting and overall context, the length, duration or type of intervention?
2. Is there evidence of effectiveness for DMT in decreasing levels of depression when pre and post-treatment scores of depression are compared?
3. Is there evidence of long-lasting effects of DMT on scores of depression?
4. Is there evidence of effectiveness when DMT is compared with no treatment, treatment as usual (TAU) or another treatment?
5. Is there evidence of effectiveness of DMT in the treatment of depression based on studies with high quality, i.e., low risk of bias?

### Objectives

- To synthesize results from all studies of effectiveness of DMT for clients with depression
- To establish effect sizes within groups, comparing pre and post scores on depression immediately after treatment and at the time of follow up
- To establish effects sizes between groups, comparing end scores between the experimental and control groups for all RCT designs and for those with low risk of bias.

## Methods

A systematic literature review was chosen as the best way to answer the main research question and as the most highly valued methodology of synthesizing evidence from different studies. According to Higgins et al. (2017), a systematic review offers a high level of evidence regarding the effectiveness of an intervention.

In this review, conventions and processes used in Cochrane reviews (Higgins et al., 2017) were adopted; the study by Meekums et al. (2015) in particular was an important reference point. However, unlike the restrictive inclusion criteria of Cochrane reviews, a more open approach to the choice of studies was followed, aiming to offer a more comprehensive picture of available evidence on the topic. In this review, both a qualitative meta-synthesis and a quantitative meta-analysis of the reviewed studies are provided. While the former retains a narrative character, the latter involves the use of statistical calculations that enable a quantitative synthesis of data from several studies.

**Figure 1:**
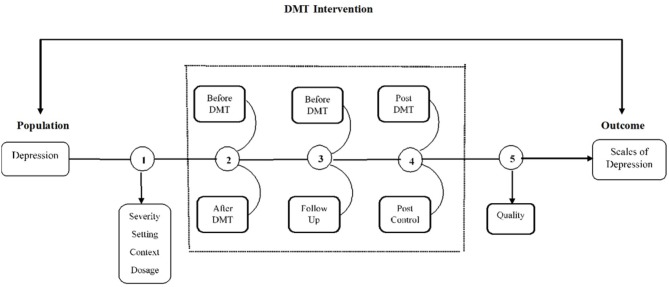
Analytic framework.

The analytic framework and its alignment with the main and sub-questions of this review is presented in Figure 1.

As indicated in this diagram, a qualitative meta-synthesis was conducted (see no. 1 in Figure 1) to answer the first sub-question of the study (i.e., “What patterns emerge from the collected evidence relating to the severity of depression, the setting and overall context, the length, duration or type of intervention?”).

As an exploratory study, a few meta-analyses were also performed to answer the four remaining sub-questions as follows:

The first meta-analysis was conducted in response to the second question (i.e., “Is there evidence of effectiveness for DMT in decreasing levels of depression before and after treatment?”) and focused on pre/post treatment scores of depression for all the studies identified by the systematic review process and synthesized through the qualitative meta-synthesis (see no. 2 in Figure 1). Long lasting effects were considered through a subgroup analysis of this initial set of studies in which only studies with follow up scores on depression were included (see no. 3 in Figure 1). The third sub-question was considered in this calculation (i.e., “Is there evidence of long-lasting effects of DMT on scores of depression?”).

The third meta-analysis summarized the effect size for RCTs only (i.e., “Is there evidence of effectiveness when DMT is compared with no treatment, TAU or another treatment?” no. 4), while the final calculation involved sensitivity analysis of this last set of studies, retaining only RCTs with low risk of bias (no. 5). Results from this analysis answered the final question of the study which was “Is there evidence of effectiveness of DMT in the treatment of depression based on studies with high quality, i.e., low risk of bias?”

### Criteria for Considering Studies for This Review

#### Study Design

All RCTs were considered as well as studies with quasi-randomization or systematic methods of allocation. Unlike the Cochrane review on DMT for depression (Meekums et al., 2015) however, in this review, controlled trials and studies with pre- post-testing were also included. Qualitative studies were excluded because they were perceived as providing information about process rather than outcome; the latter being the main focus of this review.

#### Participants

Included studies offered interventions to people with symptoms of depression as defined by the trialist and assessed through diagnostic means such as ICD-10 or DSM or through using a standardized measure such as Becks Depression Inventory, the Symptom Check List-90-Revision or the Hamilton Rating Scale. There was no restriction in terms of severity of depression, age, gender or ethnicity. Studies with participants whose primary diagnosis was something other than depression and/or individual symptoms of depression (e.g., low mood) in the absence of sufficient evidence to form a firm diagnosis of depression were excluded.

#### Intervention

The intervention was facilitated by a practitioner who had received formal training, was a dance movement therapist in training or was otherwise accredited in the country in which the study was conducted. In countries where professional accreditation was not available, the available description of the intervention was examined to establish that it demonstrated key relevant characteristics of DMT practice. DMT practice was defined as an active engagement of participants in dance movement in the presence of a therapist. All DMT approaches were considered, but in all cases the intervention had a clear psychotherapeutic intent and fostered a psychotherapeutic relationship. Dance classes with therapeutic benefit were therefore excluded.

#### Comparators

Studies with all types of comparators to DMT as a main intervention were included such as waiting list, TAU, another psychological therapy, pharmacological interventions, physical interventions or different types of DMT. In this review, and unlike the Meekums et al. (2015) review, studies without a comparator were also included.

#### Outcome Measures

Scores on levels of depression were seen as the primary outcome measure. Both self-rated standardized measurements (e.g., the Beck Depression Inventory, Beck et al., 1961; the Symptom Check List-90-Revision, SCL-90-R, Derogatis, 1977) as well as observational tools (e.g., the Hamilton Rating Scale for Depression, HAM-D, Hamilton, 1960) were considered. Attrition rates, where available, were also considered as a sign of acceptability of the intervention.

Secondary outcomes included social and occupational functioning, quality of life, self-esteem, body image, cost effectiveness and adverse events. In this paper however, only results from primary outcomes are reported.

### Systematic Review Protocol

According to Uman (2011), the presence of a protocol offers a rigorous a priori process that minimizes selection bias. The protocol for the completed Cochrane review on the topic was published by the Cochrane Collaboration (Meekums et al., 2012). As indicated before, there were a few differences from this protocol, the main being in the inclusion of types of study design. In a departure from the initial protocol (Meekums et al., 2012) that was used to guide the subsequent Cochrane review (Meekums et al., 2015), the current review included all studies with randomized, controlled and pre/post quasi experimental designs. A revised protocol was therefore prepared.

### Search Strategy

The search took place in two phases. The first was part of the Meekums et al. (2015) Cochrane review and was up to date on the 2nd October 2014.

A new search was completed between 2nd October 2014 and 1st March 2018 using the same key words and databases as the first search (see Table 1). In this second search the online package Covidence (www.covidence.org) was used which was not available at the time of the first search.

**Table 1:** Databases and search terms.

**Databases**
The specialized register (CCDANCTR-Studies and CCDANCTR-References) World Health Organization's International clinical trials registry platform (WHO) ClinicalTrials.gov Allied and Complementary Medicine Database (AMED) Education Resources Information Center (ERIC) and Dissertation Abstracts (to August 2013) Also: contacted DMT experts from around the world.
**Keywords**
Depress or dysthymia^*^ or adjustment disorder^*^ or mood disorder^*^ or affective disorder^*^ affective symptom^*^ AND Dance^*^ or authentic movement^*^ or movement therapy^*^ or movement psychotherapy^*^ or body psychotherapy

In the first instance, all known DMT professional associations were contacted through the use of a standardized letter with a request to provide any studies known to them. During the second search, key researchers in the field were contacted to provide any additional new research studies completed since October 2014.

### Data Screening, Eligibility and Data Extraction

With regards to the first set of studies identified during the Cochrane review process we revisited all studies that were excluded on the basis of the study design as indicated on the Preferred Reporting Items for Systematic Review and Meta-Analysis (PRISMA) diagram of this original review (Meekums et al., 2015).

During the second phase, all new studies found since the 2nd October 2014 were screened at a title and abstract level and then at a full text level.

As for the first phase, two reviewers were involved simultaneously while a third reviewer was on call in case of disagreements^4^. The process and the number of included studies were recorded on a new PRISMA diagram that collated both the original and the new search (see Figure 1).

In consultation with the review team, a spreadsheet was created in the Covidence software to collect and organize all the relevant information from the studies. The first two authors (VK and SA) extracted data independently from all included studies on the characteristics of the design, the population, intervention and outcomes. Effect size data (mean, SD, and number of participants) for the calculation of meta-analysis was also extracted. Any mismatches between the two sets of data-extractions and discrepancies were resolved through consensus after jointly checking the full-text papers. In case of missing data or incomplete information, VK contacted the authors via email. Permission to include one of the studies that was still unpublished at the time of the review was sought from the authors. Additional information was also requested in order to provide clarification on the methods and procedures followed and complete the assessment of risk of bias.

### Assessment of Risk of Bias

The risk of bias for all the reviewed studies was assessed using Cochrane criteria (Higgins et al., 2017): (i) random sequence generation (ii) allocation concealment (iii) blinding of participants and personnel (iv) blinding of outcome assessment (v) incomplete outcome data (vi) selective reporting and (vii) other sources of bias.

### Data Analysis and Synthesis

In the present study, careful qualitative synthesis was conducted for all studies that met the inclusion criteria. The key areas of interest for this qualitative meta-synthesis were: the severity of depression, the setting and overall context, the length, duration or type of intervention.

The quantitative meta-analyses were conducted using the Review Manager software (RevMan 5.3). The first included all studies and a within-groups calculation. The second involved studies with follow up measures. They were sub-grouped and effect size was calculated considering any long-lasting effects for DMT with this client population. The third calculation included only studies with RCT designs and between-groups scores. Finally, studies with low risk of bias were included in the last calculation that involved a sensitivity analysis.

Because of the outcome measures of depression used, the data collected was continuous. For this reason, and assuming that they measured the same construct, outcome measures of depression, such as BDI and HAM-D, were brought together for the calculation of our meta-analysis. Still, given that there were different scales used in each case, Standardized Mean Differences (SMD) were chosen over Mean Differences (MD). The SMD was calculated using Hedges' g method. This method can accommodate for the danger of a small sample size bias (Deeks and Higgins, 2010).

A random effects model (DerSimonian and Kacker, 2007) was considered as an appropriate approach for this meta-analysis. Its selection was based on the assumption that the data for meta-analysis was drawn from a hierarchy or variety of population whose differences influenced the analysis. The random effects model assumes that the included studies are not identical, and the true effect size varies between studies or there is a random distribution of true effects. This is unlike the fixed effects model which presupposes that the effects are identical (Borenstein et al., 2010).

Although there was heterogeneity, the studies reviewed in this study were reasonably comparable as all participants had a common diagnosis of depression and received the same intervention. Still, if one were to ignore methodological heterogeneity, there would be a risk of an overly precise or too narrow confidence interval summary result which may wrongly imply that a common treatment effect exists when actually there are real differences in treatment effectiveness across studies (Thompson and Sharp, 1999). Hence, to allow for unobserved heterogeneity (differences in instruments or across units being studied), a random effects meta-analysis appeared to be the appropriate analytic choice.

The overall mean or pooled estimate was calculated as a weighted average. In a random effects model, the weight is the inverse of the variance capturing the two sources of variance, within study variance and between study variance, which depends on the distribution of the true effects across studies (tau square).

Although data from secondary outcomes were also measured in some of the included studies such as anxiety, quality of life and body image, in this paper our focus remained on results from the primary outcome only.

For this paper we also considered the type of subgroup analysis performed in the Meekums et al. (2015) study based on age, but chose not to pursue this mainly due to the fact that only one study survived scrutiny that did not involve adults; this one study was also assessed as having high risk of bias. Subgroup analysis for the type of intervention was not performed either because there were no obvious differences between DMT practices used in the reviewed studies. Finally, although there were differences in the severity of depression at the start of the study, subgroup analysis on the level of depression was not performed because of the limited number of studies; differences were discussed in the narrative meta-synthesis.

## Results

### Study Characteristics and Qualitative Meta-Synthesis

**Figure 2:**
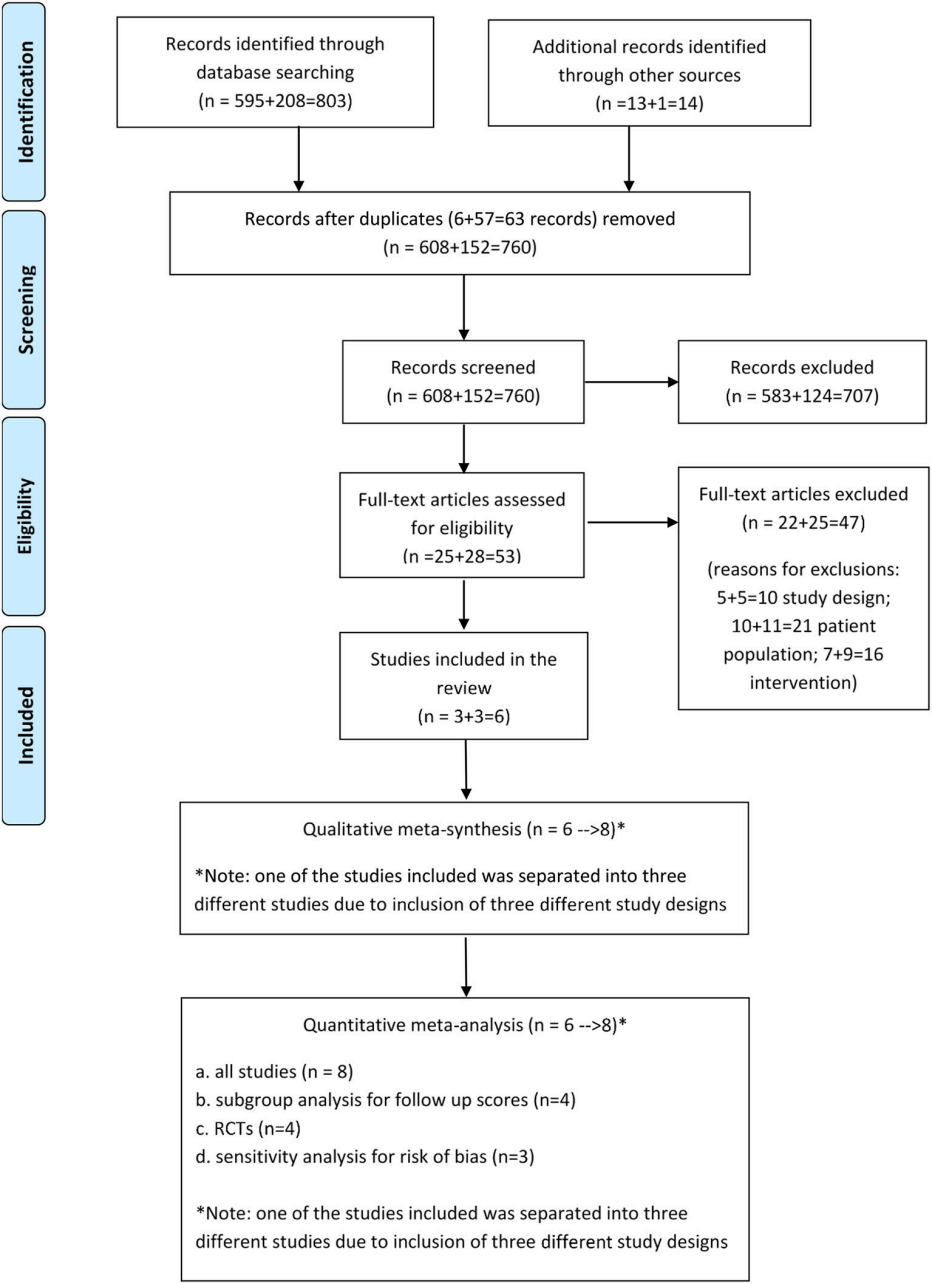
PRISMA 2009 flow diagram.

As the PRISMA diagram indicates (see Figure 2), 803 records were identified through searching electronic databases (595 were identified in the initial Cochrane review search and 208 in the newest search), whilst 14 were found through personal contact (13 during the first stage of the search and 1 in the second search), taking the total number of records identified to *N* = 817. From these records, 63 were duplicates and were taken out, leaving 760 records to screen at a title and abstract level. From these, 57 records were examined as full text articles, excluding 51 studies due to: the study design (*n* = 10), the population (*n* = 21), and the intervention (*n* = 16).

From the six studies that met the inclusion criteria, one by Hyvönen et al. (2018) was considered as three separate studies for the purposes of both the qualitative meta-synthesis and the quantitative meta-analysis because the researchers had adopted three different research designs, namely RCT, non-RCT (controlled trial) and pre- post-testing (see Figure 2 and Table 2). This study, the most recent and largest of the studies included in this review, was still unpublished at the time of writing this paper. Information about the methodology and preliminary results were supplied directly by the researchers.

**Table 2:** Study characteristics.

**Study ID**	**Sponsor**	**Location**		**Study design**			**Population**				**Interventions**				**Outcome measures**
		**Country**	**Setting**	**Method**	**Experimental**	**Control**	**Total number of participants**	**Age**	**Gender**	**Severity of depression**	**Theoretical orientation**	**No. of sessions**	**Frequency of sessions**	**Duration of sessions**	
Jeong et al., 2005	Supported by Wonkwang University, Korea	Korea	Middle school	RCT	DMT	Waiting list	40 (exp 20 and con.20)	Mean 16	40 females	Mild	Groups designed around four major themes: (a) awareness of the body, the room, and the group; (b) movement expressions and symbolic quality of movement; (c) movement, feeling, images, and words; and (d) differentiation and integration of feelings	36	3 times a week	45 min	SCL-90-R (depression scale DEP and SOM, O–C, I–S, ANX, HOS. PHOB, PAR, PSY and global scores: GSI, PST, and PSDI). Plasma serotonin, dopamine and cortisol
Xiong et al., 2009	Not available	China	Hospital	RCT	DMT plus Treatment as Usual (TAU)	TAU	76 (exp.38 and con.38)	Mean 32.26, SD 8.71	33 males and 43 females	Very severe	Group informed by Chace methods and analytic psychology	20	5 times a week	120 min	HAM-D Chinese version of GSES
(Röhricht et al., 2013) (also, Papadopoulos and Röhricht, 2014)	No external funding	UK	Adult outpatients in secondary mental health services	Cross-over RCT	Body Psychotherapy in Chronic Depression delivered by a dance movement therapist, plus TAU	TAU	31 (exp.15 and con16)	Mean 47.7, SD 10.4, range 18-65	18 males and 13 females	Very severe with current episode of over 2 years	Manualized group designed to address symptoms of depression, with influences from body psychotherapy that included movement-based work, interactive components and insight work.	20	Twice a week	90 min	HAM-D, MANSA, Rosenberg Self-Esteem Scale
(Punkanen et al., 2014) (also, see Punkanen et al., 2017)	Finnish Center of Excellence in Interdisciplinary Music Research, University of Jyväskylä	Finland	Private center	Pre-/post-testing, pilot study	DMT plus some received medication but no other form of therapy	No control	21	Mean 40, SD 13, range 18-60	3 males and 18 females	Moderate	Group, solution focused and resource-based	20	Twice a week	60 min	BDI, HADs Anxiety, TAS, DIF, TAS, DDF TAS, EOT TAS, Total BFI, Extraversion BFI, Neuroticism RQ-A, Secure RQ-B, Fearful RQ-C, Preoccupied RQ-D (Attachment), Dism Satisfaction with Life Scale (SWLS)
(Pylvänäinen et al., 2015) (also, see Pylvänäinen, 2018; Pylvänäinen and Lappalainen, 2018)	Support from City of Tampere Psychiatric Clinic	Finland	Psychiatric outpatient clinic	Controlled Trial	DMT plus TAU	TAU	33 (exp.21 and con 12)	Mean 41, SD 11.9, range 20–59	9 males and 24 females	Moderate or severe, recurrent and/or chronic type	Group following the interactive model of Chace and analytic psychology of Authentic Movement	12	Once a week	90 min	BDI-II, HADs Anxiety, SCL-90, CORE
(Hyvönen et al., 2018) RCTs	The Finnish Social Insurance Institution (KELA)	Finland	Different settings	Multi-centered RCT (cross over design) Randomized groups in five larger cities	DMT plus TAU	TAU	109 (exp. 52 and con. 57)	Mean 42, range: 18–64 For all three strands	5 males and 145 females for all three strands	Moderate	Group, Chace model and Authentic Movement	20	Twice a week	75 min	BDI CORE-OM SCL-90 FFMQ RQ (Attachment); WAI Body image interview
(Hyvönen et al., 2018) non-RCTs	The Finnish Social Insurance Institution (KELA)	Finland	Different settings	Non-randomized groups (controlled trial) in smaller cities	DMT plus TAU	TAU	36 (exp. 20 and con. 16)	Mean 42, range: 18–64 for all three strands	5 males and 145 females for all three strands	Moderate for all three strands	Group, Chace model and Authentic Movement	20	Twice a week	75 min	BDI CORE-OM SC-90 FFMQ RQ (attachment style) WA Body image interview
(Hyvönen et al., 2018) Disability pension	The Finnish Social Insurance Institution (KELA)	Finland	Not known	One group with pre-/post-testing with participants who received disability pension	DMT plus TAU	TAU	5	Mean 42, range: 18–64 for all three strands	5 males and 145 females for all three strands	Moderate for all three strands	Group, Chace model and Authentic Movement	20	Twice a week	75 min	BDI CORE-OM SC-90 FFMQ RQ (attachment style) WA Body image interview
	Funded-3 Support from the University or clinic-3 Not funded-1	Finland-5 Korea-1 China-1 UK-1	School-1 study Different settings-2 Hospital (OPD-1) (IPD-1)	RCT- 4 studies Controlled-2 Pre- Post- 2	DMT plus TAU-6 Body Psychotheray-1 DMT-1	TAU-6 Waiting list-1	Range: 5–109	Range: 16–64	Two hundred and eighty three Females(81% out of the total participants) Sixty eight Males (19% out of the total population)	Range- Mild to Very Severe	Group, Chace model and Authentic Movement- Most frequent Manualized protocol- 1 study	Range:12–36	Range: 1 session/ week to 5 sessions per week Two sessions/ week-5 studies Five sessions/ week-1 study Three session/ week-1 study One session/ week-1 study	Range: 120–45 min 120 min-1 90 min-1 60 min-2 75 min-3 45 min-1	BDI (two versions) HAM-D and SCL

Similarly, there was more than one reference associated with some of the studies included, e.g., Pylvänäinen et al. (2015), Röhricht et al. (2013) and Punkanen et al. (2014) leading to the inclusion of eight studies from 817 records identified (Table 2).

#### Sample Size

A total of 351 participants were involved in all the studies included in this review (Table 2). From those participants, 192 attended DMT groups and 159 were part of control groups.

#### Study Design

Methodologically the eight studies ranged from pilot studies with pre/post testing (Punkanen et al., 2014), to a controlled trial (Pylvänäinen et al., 2015), three small RCTs in one location and with one therapist in each (Jeong et al., 2005; Xiong et al., 2009; Röhricht et al., 2013) and a triple multi-centered study involving several therapists that followed three types of designs: randomization in some locations, non-randomization in some others and pre/post testing in a third (Hyvönen et al., 2018). Both the Röhricht et al. (2013) and the Hyvönen et al. (2018) RCT strand had a cross over design.

#### Setting

Although the studies came from different parts of the world such as Korea, China, and the UK, it is worth noting the increased research activity in Finland with three studies completed there, one of which (Hyvönen et al., 2018) had three different strands that were conducted in different settings and different cities across Finland. The other two studies from Finland (Punkanen et al., 2014; Pylvänäinen et al., 2015) were conducted in an outpatient psychiatric clinic and in a private center. Two of the remaining studies took place in a psychiatric/mental health hospital either as inpatient provision (Xiong et al., 2009) or as an outpatient community service (Röhricht et al., 2013). The oldest of the reviewed studies took place in a middle school in Korea (Jeong et al., 2005).

#### Participants

There were 68 male and 283 female participants, the latter being 81 percent of the total population. One study, the Korean (Jeong et al., 2005), recruited exclusively female participants. The studies in Finland were mixed but involved more women than men. In two of the included studies, the Chinese (Xiong et al., 2009) and the UK (Röhricht et al., 2013) studies, the ratio between men and women was balanced with 51 men and 56 women participating in the two studies.

With the exception of the study by Jeong et al. (2005) which involved adolescents, all of the included studies addressed adults with depression. The age range for the studies with adults was 18–65 with an average of 40.6 years of age. The average age for the Korean study was 16 (Jeong et al., 2005).

#### Interventions

As Table 2 shows, the number of sessions varied from 12 in the Pylvänäinen et al. (2015) study to 36 in the Jeong et al. (2005) study with all the remaining studies offering 20 sessions. DMT groups were offered once, twice, three times and, in the case of the Chinese study, five times per week. The duration of each session also varied from 45 min in the Korean study that took place in a school to 120 min in the Chinese study that took place in an inpatient psychiatric hospital. The studies conducted in Europe included sessions that lasted from 60 to 90 min each.

In three of the studies reviewed here, the models of DMT adopted were named, and to varying degree described, as a combination of the interactive model by Chace with influences from Authentic Movement and analytic psychology (Xiong et al., 2009; Pylvänäinen et al., 2015; Hyvönen et al., 2018). From the remaining studies, one offered a description of themes that resembled a Chacian approach to DMT practice (Jeong et al., 2005), another conceptualized the intervention with strong influences from solution-focused and resource-based to reflect the additional training of the therapists involved in the study (Punkanen et al., 2014), while the last labeled the intervention as Body Psychotherapy and offered a detailed description of the intervention that resembled a manualized version of DMT practice (Röhricht et al., 2013). This last study was also delivered by an experienced DMT practitioner.

From the included studies, there are several detailed descriptions of the intervention offered through separate publications (Papadopoulos and Röhricht, 2014; Punkanen et al., 2017; Pylvänäinen, 2018).

In the studies where there was a control group, DMT groups were added to existing TAU and were compared with TAU alone. The only exception to this was the Korean study, for which the control group was a waiting list. TAU often included medication and some brief contact with a mental health professional. Participants in all the studies included did not receive another form of regular and weekly psychotherapy.

#### Outcomes

In all studies the primary outcomes were the severity of depression measured through SCL, BDI, and HAM-D. Two different versions of BDI were used (the first one published in 1961 and the other a revised version BDI II published in 1996). BDI and SCL are self-reported inventories, while HAM-D is observational. Despite their differences, these tools are regarded as sensitive to capture mood, body image, health anxiety, sleep loss, appetite and many other factors related to the diagnostic criteria of depression.

**Figure 3:**
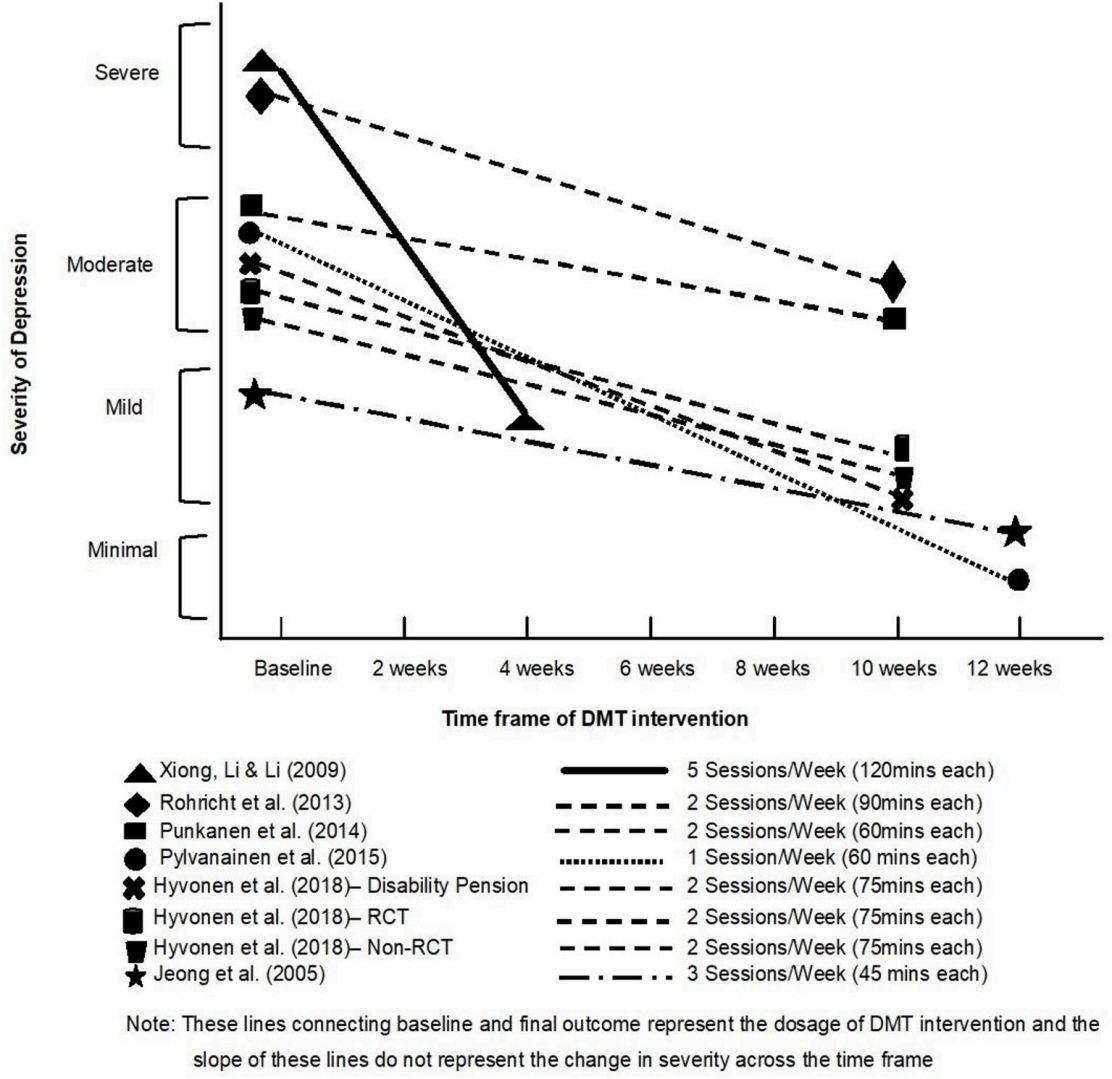
Depression outcome scores before and after DMT groups.

As shown on Figure 3, all the included studies showed a decrease in the severity of depression. Two studies (Xiong et al., 2009 and Röhricht et al., 2013) involved participants with very severe depression and the majority of the other studies (all five Finnish studies) involved participants with moderate depression at the beginning. Only the Jeong et al. (2005) participants had mild depression at baseline assessment. Toward the end of the DMT intervention, all the studies with moderate severity of depression at baseline showed a reduction to either mild (Punkanen et al., 2014; Hyvönen et al., 2018) or minimal depression (Pylvänäinen et al., 2015). Results from the randomized and well-controlled Röhricht et al. (2013) study indicate a gradual shift from severe depression to moderate depression. Xiong et al. (2009) report a drastic improvement and a sharp shift from severe depression to mild depression. Pylvänäinen et al. (2015) is the only study which resulted in participants having minimal depression after the DMT intervention, i.e., appearing to present full recovery.

## Risk of Bias

**Figure 4:**
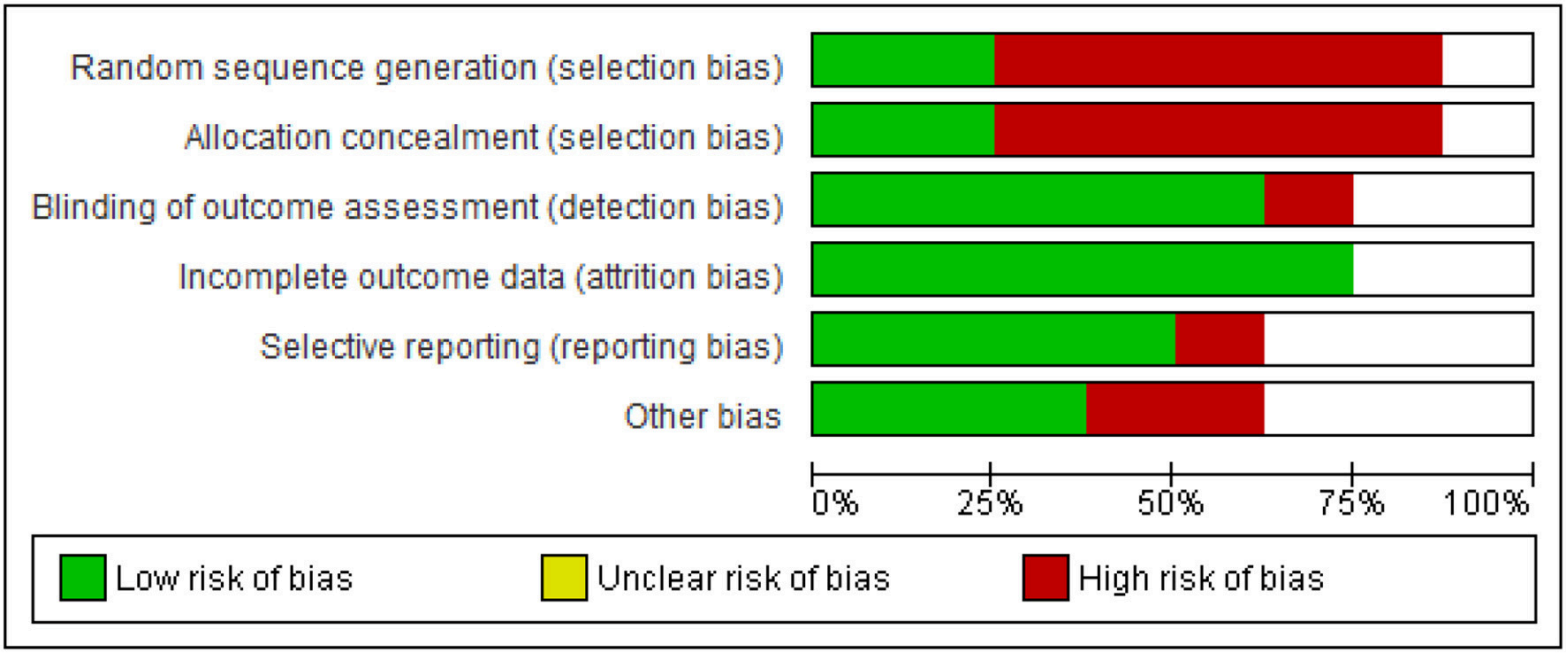
Risk of bias across all included studies.

**Figure 5:**
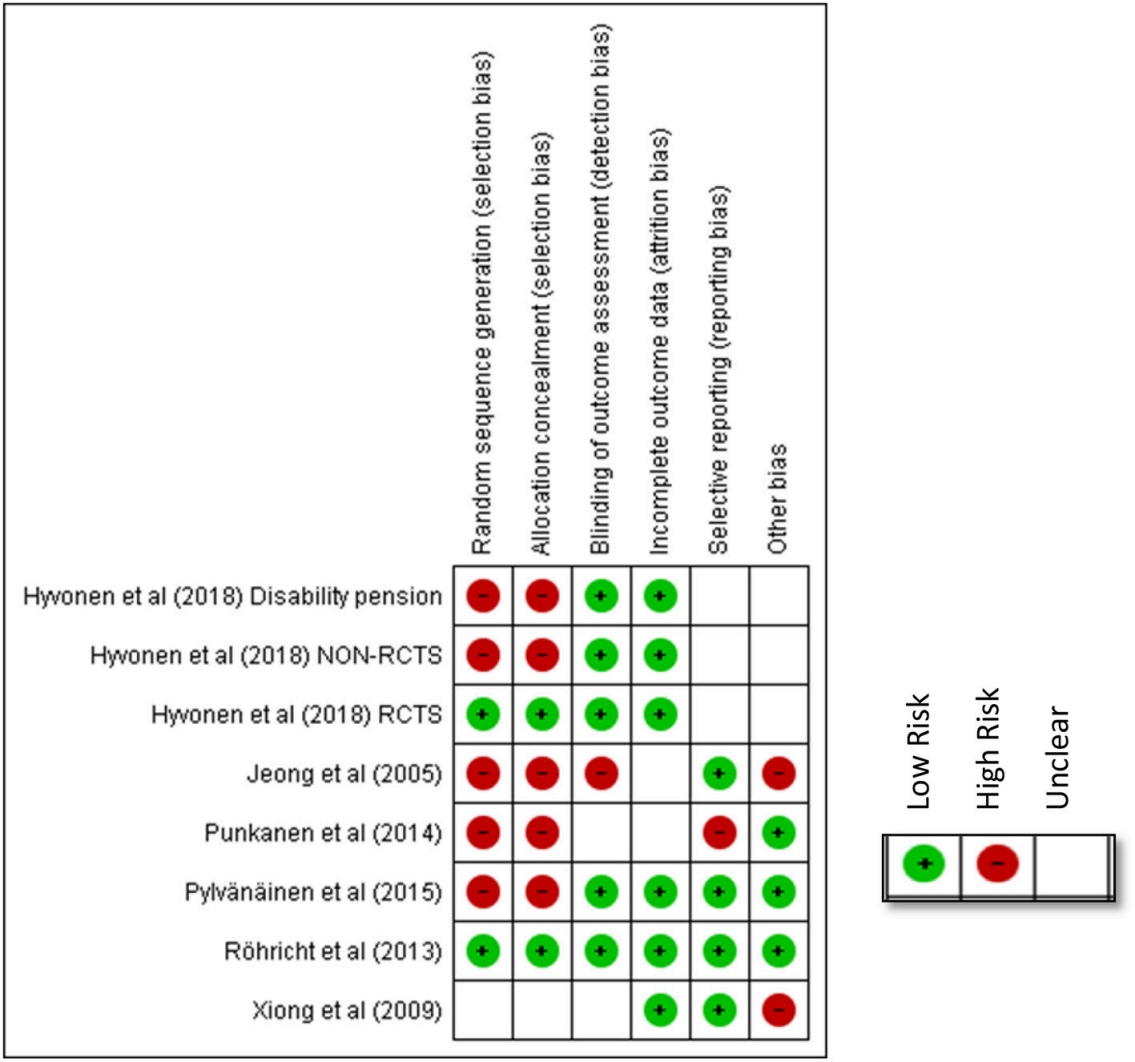
Risk of bias for each included study.

Figures 4, 5 shows the risk of bias of the studies assessed against Cochrane criteria (Higgins et al., 2017). An emphasis on randomization and blinding is included in the risk of bias assessment. However, in three of the eight studies, randomization had not taken place resulting in high risk of bias (Punkanen et al., 2014; Pylvänäinen et al., 2015; Hyvönen et al., 2018 disability pension group). Furthermore, blinding for participants and personnel, as for all studies in psychotherapy, was not possible. For this reason, this criterion was omitted from the assessment of quality as suggested by Schünemann et al. (2013).

As Figure 5 shows, the earliest included publication, the study by Jeong et al. (2005) had quality limitations, even though an RCT design was followed. Only one of the risk of bias criteria was scored as low, namely selective reporting (see green color). Most of the remaining criteria were scored as high risk (red color) or uncertain risk (empty box).

The study from China, Xiong et al. (2009), was of moderate quality, presenting concerns due to insufficient information around random sequence generation, allocation concealment and blinding of outcome assessment. Attempts to contact the authors to clarify these were unsuccessful. Since this study was the only study not published in English^5^, it is possible that the language created a barrier that we did not manage to overcome.

The study by Röhricht et al. (2013) was assessed as the study with the highest quality (the lowest risk of bias). Both the design and a thorough reporting against all criteria of risk of bias added to the quality of study.

In contrast, the study by Punkanen et al. (2014) had methodological limitations mainly due to the fact that it did not have a control group and thus, there was no randomization. In addition, since this was a small pilot study the researchers tried different methods, the outcomes for all of which were not reported, including results from measurements of attachment styles. From the findings presented, it was not clear whether outcome assessors were blinded for all outcomes. It was not clear either how many participants were involved, and information concerning attrition was omitted. However, this was a multi-faceted study presenting rigor and clarity of roles between the research team from the University of Jyväskylä and the team of therapists involved. The first phase of the study reported in Punkanen et al. (2017) also adds to the DMT literature in that through the use of the technology “motion capture” it outlined movement characteristics particular to people with depression; comparisons were made between a group of people with depression and a group of people with no depression identifying important movement differences between the two groups.

The study by Pylvänäinen et al. (2015) is a study “in the real world.” The limitations that our risk assessment highlights are compensated for the fact that the study was conducted in a clinical environment and as part of regular work. The findings are thus potentially directly applicable to clinical work.

The value placed on randomization in the conventional hierarchy of evidence (Higgins et al., 2017) is at odds with the prevalent culture in a clinical setting that prioritizes client choice. While the Cochrane criteria concerning risk of bias imply that the therapist should be a different person from the researcher, the dual role in practice might add an element of trust, rigor and depth both for the development and delivery of the intervention and for an insightful interpretation of results (Meekums, 1998).

Finally, in the design of the study by Hyvönen et al. (2018) there is strong potential to compensate for the risks of bias in all previous studies without limiting the quality of the intervention. However, since this study had three different strands with different designs (RCTs, controlled trials and pre/post testing), the risk of bias in these strands was different. Furthermore, given that findings were still being processed at the time of writing this review, we were unable to include information for all the criteria each study was assessed as indicated on Figure 5.

To summarize our findings, as indicated in Figure 4, 75% of the included studies had low risk of attrition bias. This is the only criterion which most of the studies met. The next lowest risk of bias criterion was detection bias. Whilst as for all types of psychotherapy, it is impossible to blind participants to the type of intervention, it appears from our results to be less challenging to blind for the outcome assessment. The type of measuring tools used in the study (observational/self-reports) might have played a role in allowing (e.g., in the case of observational measures) or hindering (e.g., in the case of self-rating scales) the possibility for blinding for the outcome measure. Since, we have included quasi-experimental designs it is obvious that only 25% of the included studies had low risk of bias in sequence generation and allocation concealment and the majority (75%) of the studies were therefore, of high risk.

## Quantitative Meta-Analyses

Meta-analyses were performed as a way of synthesizing quantitative evidence of effect size across studies. Although studies varied in their designs, they all addressed the same fundamental question around the effectiveness of DMT. These different designs were therefore grouped in different ways in order to identify the direction of effect and effect size. The analyses performed were based on four different data sets as follows:

- Studies with before and after DMT scores; all the reviewed studies were included
- Studies with 3 months follow up data (pre DMT vs. 3 months follow up) as a sub-group analysis
- Studies with RCT designs only (post DMT experimental vs. post TAU or no care)
- Studies with RCT designs with lower risk of bias through sensitivity analysis.

**Figure 6:**
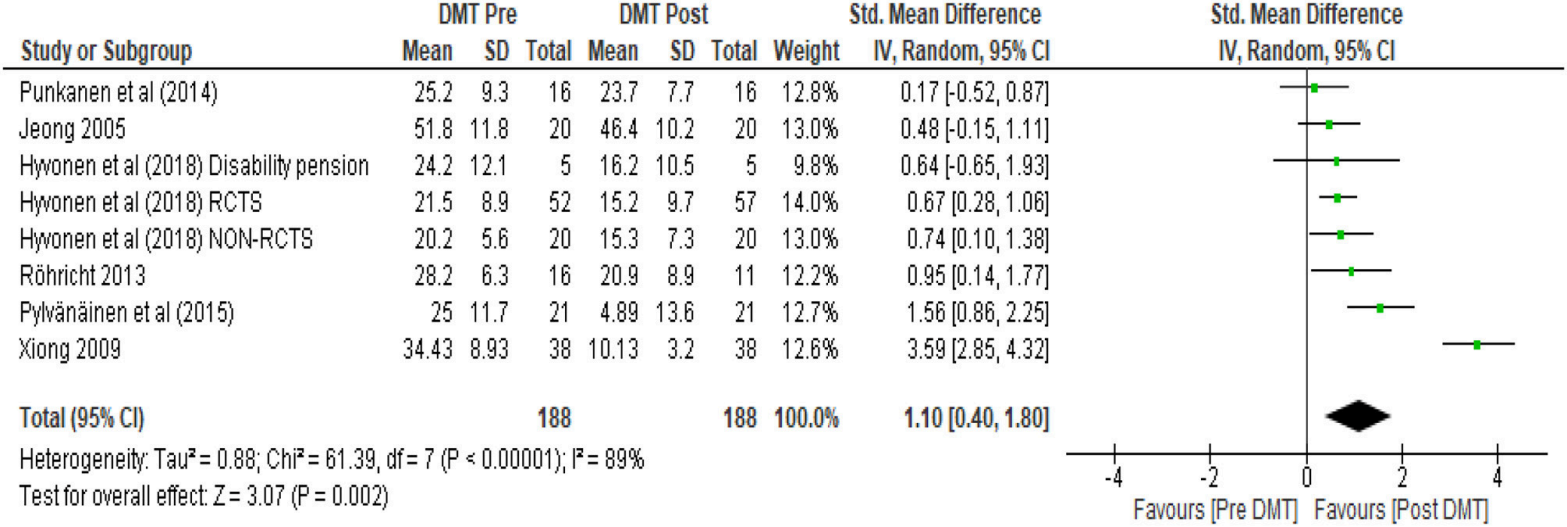
Within DMT groups (pre DMT vs. post DMT).

**Figure 7:**
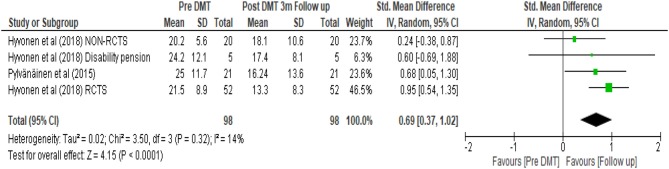
Subgroup analysis for within DMT groups (pre DMT vs. 3 months follow up).

**Figure 8:**
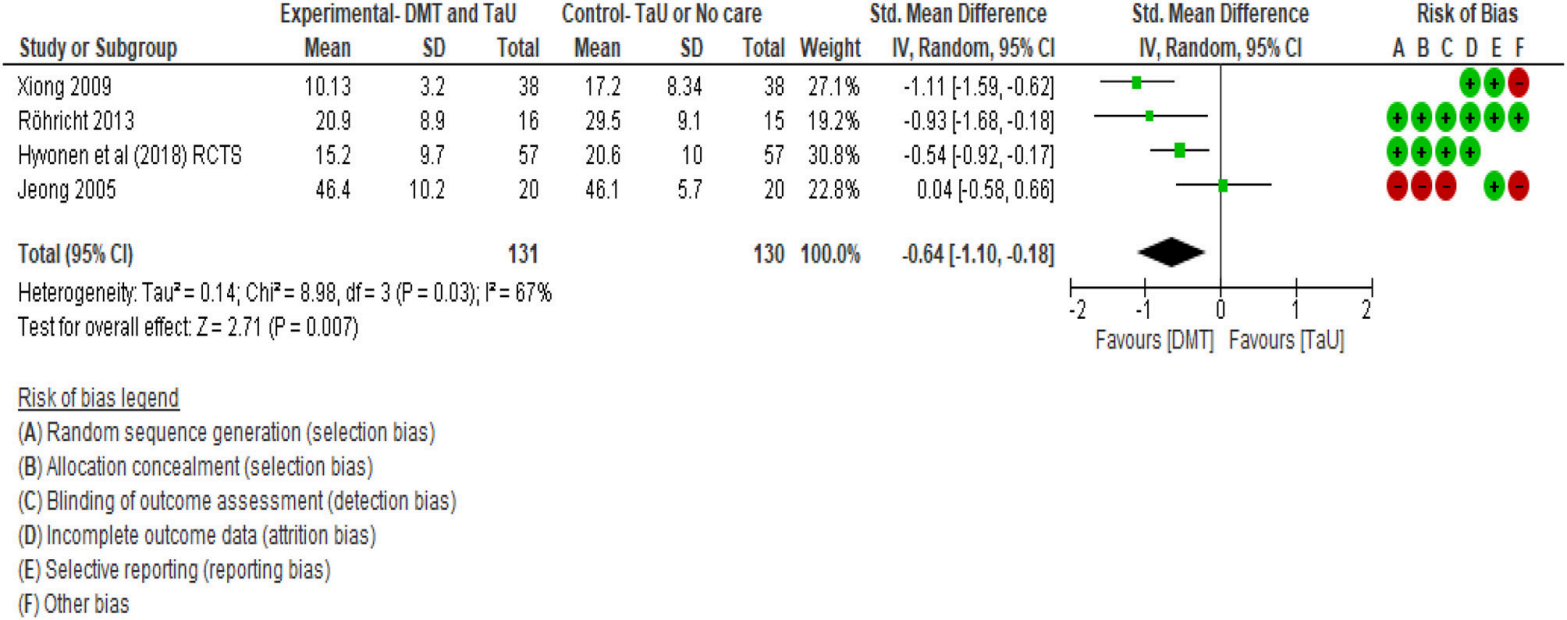
Between Groups—RCTs (post DMT vs. control).

**Figure 9:**
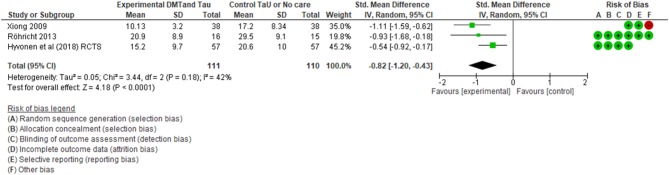
Sensitivity analysis for between groups—RCTs only with moderate to low risk (post DMT vs. control).

As per our protocol, only scores until the point of the crossover were considered. In all four cases the effect of DMT in decreasing scores on depression was evaluated. As shown in the forest plots (Figures 6–**9**), each study included for the analysis was represented by its point estimate with 95% confidence interval. It is noticeable from the plots that the size of the square between the studies varied based on the allocated weight associated with the calculation of each power estimate. The larger studies with less variance and more precise results were given larger weight. The overall measure of effect and the direction of effect is visually represented on the forest plots by the location of the diamond.

The first analysis (see Figure 6) in which all reviewed studies were included unsurprisingly had the largest total number of participants (*N* = 188). The SMD using a random effects model was 1.10 (95% CI 0.40, 1.80). As the confidence interval did not contain zero, there was strong evidence of a positive treatment effect. The *I*^2^ was calculated which is a measure of heterogeneity amongst studies indicating the percentage of variance amongst studies (Higgins et al., 2003). In this calculation *I*^2^ was 89%, suggesting 89% of the variability in treatment effect estimates was due to real study differences (heterogeneity) and only 11% due to chance. This is visually evident from the wide scatter of effect estimates with little overlap in their confidence intervals (Figure 6). Xiong et al. (2009) showed greater effect estimate than all the other studies, appearing further apart from these other studies.

A subgroup analysis was performed on this initial set of data as shown in Figure 7. Only studies with a follow up depression score were included in order to assess any lasting effects of DMT. This calculation had the smallest number of participants (*N* = 98). The random effects model provided an estimate of the average treatment effect. It revealed 0.69 SMD with 95% confidence interval (0.37, 1.02). Using Cohen's rule of thumb (Lipsey and Wilson, 2001), a SMD of 0.69 was considered to be of medium effect. Although the confidence interval depicts uncertainty around this estimate, since the confidence interval does not cross the zero line, it shows positive effect of the treatment even 3 months after completion of the intervention.

In this calculation, the *I*^2^ was 14%, suggesting that variability in treatment effect was mainly due to chance. Regardless of treatment, people may recover in time on their own, but some may do so at a slower rate than others. The rate of their recovery may be to a great extent influenced by their baseline characteristics or condition. Thus, patient characteristics need to be considered and the findings should be interpreted with caution.

When the SMDs were compared as end scores between the groups that received DMT and the control groups, an effect size of −0.64 favoring DMT treatment was found (see Figure 8). This third calculation included all the RCTs that were found in our included studies. The total number of 131 participants were involved in four studies. The confidence interval ranging between −1.10 to −0.18 did not cross zero. This supported the effect direction favoring the DMT group. In terms of heterogeneity, ~67% of the variability in treatment effect estimates was due to real study differences among the studies and only ~33% was due to chance. Figure 8 shows that there is wide scatter of effect estimates with little overlap in their confidence intervals. Among these four RCTs, the study by Jeong et al. (2005) was the weakest study as indicated by the number of negative red marks in the risk of bias table (Figure 5), suggesting very low methodological quality. Hence, a “crude sensitivity analysis” was carried out (see Figure 9).

As shown on Figure 9, only three RCTs with moderate to low risks were included in this last calculation that included end scores from 111 participants, all of whom were adults. Our meta-analysis showed a SMD of −0.82 suggesting a large effect size according to Cohens rule of thumb (Lipsey and Wilson, 2001), favoring the DMT intervention. The confidence interval was −1.20–0.43. Since it did not contain zero, there is limited uncertainty around the average treatment effect estimate. We can therefore say with confidence that DMT has an impact in reducing scores of depression. The confidence interval depicts the uncertainty around the average treatment effect estimate. In this analysis, as the confidence interval does not contain zero, there is strong evidence that, on average, the DMT effect is beneficial to the participants.

A heterogeneity analysis with *I*^2^ calculation demonstrates that 42% of the variability in treatment effect estimates was due to between study variations and the rest due to chance. Although there were only three studies, all three were of moderate to low risk of bias and there was consistency in the findings.

Results from this final calculation demonstrate the highest level of effectiveness of DMT in treating depression amongst adults. Since we used a random effects model we have not ignored heterogeneity and thus, we have a precise confidence interval (Brockwell and Gordon, 2001; Borenstein et al., 2010). So, it is less likely that our summary result may wrongly imply that a treatment effect exists when actually there are real differences in the effectiveness of treatment across studies.

## Discussion

### Summary of Main Results

This review gathered evidence from eight studies that involved 351 participants. Unlike the Cochrane review of DMT for depression (Meekums et al., 2015), in this new review we were able to be quite conclusive concerning the effects of DMT on depression: when we conducted a sensitivity analysis on three studies of moderate to high quality involving 111 participants, we found that DMT offered in addition to TAU had the highest impact on decreasing levels of depression compared to TAU. This result is in accordance with a previous but differently focused meta-analysis that suggested dance and DMT can be potentially effective in decreasing symptoms for depression (Koch et al., 2014). Through this review, we can now say with confidence that DMT exclusively (that is, without combining results of dance and DMT studies together) can have a positive impact on patients with a primary diagnosis of depression.

Interestingly, when scores from all RCT designs were considered in our calculations, including studies with high risk of bias, a smaller (i.e., moderate) effect size was found, suggesting that the lower quality studies dropped the calculated effect size.

The meta-analysis performed with all studies and with all designs on scores of depression before and after the DMT interventions also indicate a favorable trend for DMT groups since scores of depression were decreased in all cases. Since scores from control groups were not considered in this calculation, it is not possible to know whether this change took place due to what Eysenck (1963) termed “spontaneous remission.” We therefore, do not know whether participants simply recovered because time passed as opposed to the beneficial effect of DMT.

Similarly, following from arguments in the literature that DMT is not simply a form of dance and/or exercise that provides temporary relief only (Karkou and Sanderson, 2006; Meekums et al., 2015), we explored whether any long-lasting effects could be found. Calculations with studies that had included follow up measures resulted in a moderate effect size, which however was not conclusive due to different baseline characteristics of the participants and other variables influencing the results. The heterogeneity found in these studies was majorly due to chance, suggesting the need for further research attention in this direction.

Although on the basis of both of these two last calculations, we are not able to draw firm conclusions, we can see certain trends which can have useful clinical and research implications. For example, in all cases we can see that the scores on depression decreased, and this decrease continued several months after the completion of DMT.

### Gender

Another important trend relates to the gender of participants. The fact that 81 percent of the participants were women may reflect the fact that dance is seen as an art form that stereotypically attracts women. Research bias can be seen in studies such as Jeong et al. (2005) that involved adolescent girls only. When choice was offered as was the case with most of the remaining studies, most studies, with the exception of Xiong et al. (2009) and Röhricht et al. (2013), did not accommodate for gender diversity resulting in samples with a large number of women (see all the Finnish studies for example). This skewed sample limits our capacity to draw firm conclusions that DMT can be of equal value to both men and women, especially since there is research literature to suggest that men and women respond differently to the use of psychotherapy (Ogrodniczuk, 2006).

### Age

Unlike gender, the age of our sample was widely ranging from 16 to 65 years of age. However, all studies but one (Jeong et al., 2005) did include adults with depression only. Although it was not our intention to focus solely on adults, the evidence we found related to the effectiveness of DMT mainly with this age group. Nevertheless, as reported in several publications before (Karkou and Sanderson, 2006; Karkou, 2010; Karkou et al., 2010), dance movement therapists work extensively with children and adolescents. However, Zubala and Karkou (2018) argue that depression is rarely diagnosed amongst children and adolescents. This could explain why we only have one study included in this review that involved a non-adult population.

Similarly, studies with people older than 65 who may be struggling with depression were also missing despite the increased research activity relating to people in this age group (Karkou and Meekums, 2017; McHitarian et al., 2017). Co-morbid medical conditions such as dementia, Parkinson's, heart disease, strokes and so on might explain why studies with depression as a primary diagnosis were not found.

### Severity of Depression

As indicated in our qualitative narrative synthesis and our first meta-analysis, all the studies included in this review demonstrated a decrease in the levels of depression for the intervention with participants with a range of levels of depression. As Zubala and Karkou (2015) suggest, dance movement therapists work with clients with depression extensively, some of whom are fairly unwell, presenting moderate or severe levels of depression. As expected, when the work took place in hospitals and in psychiatric units, the severity of depression was higher than in other settings as we see in the different baseline scores in studies by Xiong et al. (2009) and Röhricht et al. (2013). Most of the participants on average had moderate depression (in 5 out of 8 studies) at baseline which was reduced to mild in most of the cases. The only study where participants had mild depression at baseline was in the Korean study that took place in a mainstream school and involved adolescent girls (Jeong et al., 2005).

### DMT Dosage

As presented in our qualitative narrative meta-synthesis, the most common trend amongst the reviewed studies was two sessions per week across 10 weeks. The only Chinese study (Xiong et al., 2009) offered five sessions per week for 120 min each time for 4 weeks, in an inpatient hospital setting. Another study with high DMT dosage was the study by Jeong et al. (2005) that offered three sessions per week. This latter study was the only study that was conducted in a school setting. It is possible that in a school environment, and in inpatient hospital environments as was the case with the Chinese study by Xiong et al. (2009), it is more feasible to have frequent sessions when compared to community-based settings. It is also worth noting that the two studies with the higher frequency of sessions were from Korea and China. It is therefore, possible that culture may have a role to play on the high treatment dosage in these two studies.

The length of the sessions ranged from 45 to 120 min. On average, and in all the European studies, sessions lasted from 60 to 90 min. The shortest sessions were available in the Korean study by Jeong et al. (2005) that offered 45 min-long sessions. The age of the participants and plans around fitting to the school timetable might be reasons to explain this choice. The study with the longest sessions was the Chinese study (Xiong et al., 2009) that offered 120 min each time; a fairly unusual length of time for a DMT session in Europe and the USA. This might be associated with either the cultural context and/or the severity of depression of the participants in this study.

On the whole, it is worth considering whether high therapy “dosage” was associated with higher level of severity of depression. In the Chinese study (Xiong et al., 2009) for example, participants with severe depression received high overall DMT dosage. There was also a dramatic decrease on the levels of depression post DMT. Although in the literature we can find arguments for the need for longer term interventions for clients with higher levels of distress (Lutz et al., 2015), the intensity of sessions in this study of five sessions per week, as far as we know, has only been seen in Freudian analysis (Freud, 1917). In DMT, and given the physical engagement of participants, it is possible that such intensity may lead to fatigue. Furthermore, given that length of this intervention was only 4 weeks, it was not clear whether underlying issues were sufficiently processed, a practice that may also lead to relapse, a common feature of cognitive behavioral therapy (Ali et al., 2017) and exercise (Sullum et al., 2000).

Another study that involved participants with similar levels of severity of depression at the baseline was the study by Röhricht et al. (2013). Even with a lower dosage, the severity of depression was still reduced, albeit less dramatically than in the Chinese study. Given the successful results from both of these studies in reducing depression to either mild or moderate, it is worth considering whether a more intense dosage of DMT is needed with severe depression.

With the exception of the study by Pylvänäinen et al. (2015), most studies that involved participants with moderate depression offered DMT groups twice a week for 20 sessions. The Pylvänäinen et al. (2015) study offered only one session per week for 12 weeks, but still demonstrated substantial changes on levels of depression. Follow up scores also indicated that the low level of depression remained 3 months after the completion of the intervention, with the participants in the study not returning to the clinic for at least 3 years after the completion of the intervention. Based on this, we speculate that a degree of time between sessions might be needed to allow for processing some of the deeper work that can take place in sessions (Karkou and Sanderson, 2006). It is also worth considering whether more frequent sessions are needed initially as in the Xiong et al. (2009) study. Once severity is reduced, the dosage of therapy might need to be gradually reduced offering DMT over a longer period, as in the Pylvänäinen et al. (2015) study, consolidating and stabilizing any acquired changes.

All the studies except for Punkanen et al. (2014) showed a shift in the level of depression. In the Punkanen et al. study (2014), there was a decrease in the scores of depression post DMT, but the level of depression did not drop from moderate to mild. Since this was a small pilot study, researchers and therapists might have tried different DMT methods and processes in the sessions.

### Type of Intervention

On the whole, the studies included in this review used integrative models that combined the interactive model by Chace (Chaiklin and Schmais, 1986) with in-depth methods developed by Whitehouse (1979). Variations to these can be found in the study by Jeong et al. (2005), where the description of the intervention is thin and relevant references are not included even if the brief description does resemble DMT practice. Similarly, the Chinese study (Xiong et al., 2009) offered thin descriptions around the intervention but named Chace and Jungian psychology as strong influences in the intervention used. Although in these two studies dance movement therapists were not used, the studies were included because the discipline in these two countries at the time the studies took place was still in the process of development and professionalization and the descriptions of the intervention included in these two studies met our DMT definition.

The DMT approaches used in the Finnish studies is worth looking at carefully. The Pylvänäinen et al. (2015) study presented a very comprehensive treatment protocol and a significant decrease in scores of depression (Pylvänäinen, 2018). Key principles from this study were also used to inform the intervention used for the large multi-centered study completed by Hyvönen et al. (2018). In both cases Chace (Chaiklin and Schmais, 1986) and Whitehouse (1979) were mentioned as important influences in the work. The third of the Finnish studies (and the first of the included studies that was conducted in Finland) by Punkanen et al. (2014) was the only study that named solution-focused and resource model as the basis for this intervention (Punkanen et al., 2017). Similarly, the UK study by Röhricht et al. (2013) indicated strong influences from Body Psychotherapy, a form of psychotherapy linked to DMT practice but less often discussed amongst DMT practitioners (Payne et al., 2014).

In all the studies conducted in Finland and the study in the UK, qualified and registered dance movement therapists delivered the intervention. Due to their training, it is possible that similar methods were used and an overall integrative model of DMT practice was adopted reflecting similar trends in psychotherapy in general (Norcross and Goldfried, 2005). This integrative approach limited our capacity to comment on whether one type of DMT practice was more relevant to depression than another or whether certain active ingredients were more “potent” than others; the whole “package” appeared to contribute to decreasing levels of depression.

### Quality of Studies

The study with the highest quality at the time of writing up this review was the UK study by Röhricht et al. (2013). This was led by a psychiatrist who offered important support to a new intervention such as DMT. It may also be significant that this study was conducted in the UK, a country where DMT has been practiced in hospitals since the 1970s; the profession is relatively established (ADMP UK, 2013) and recognized as a form of psychotherapy.

In contrast, the first review study by Jeong et al. (2005) remained of low quality and was subsequently dropped from our final calculation. Its low quality could reflect both the historical period during which the study was conducted and the professional development of DMT in that country at that time.

All three of the most recent studies came from Finland, a country with particular interest in identifying appropriate treatment for depression due to its high rate of depression and Seasonal Affective Disorder (SAD—Saarijärvi et al., 1999; Magnusson, 2000); also a country with high quality in health provision (Afonso and Aubyn, 2006). In addition, it appears that the team of the Finnish DMT researchers gradually built on evidence from a preliminary pilot stage to a large, well-funded study supported by the Finnish Social Insurance Institution (KELA), which is responsible for funding health interventions. It is possible that this last study by Hyvönen et al. (2018) benefitted from the knowledge gradually accumulated in the field and within the particular research team. Furthermore, as a multi-centered study, it was delivered by different therapists in different locations in both large and smaller studies. Because of the presence of different therapists, we can argue that significant results in decreasing the scores of depression were not based on the particular skills and/or charisma of the therapist but on the intervention itself, supporting our confidence on the beneficial impact of DMT on the treatment of depression.

## Conclusions

During this systematic review we were able to explore evidence of effectiveness around the role of DMT in the treatment of depression and answer our main research question concerning whether DMT is an effective treatment for clients with a diagnosis of depression. We conclude that there is evidence from high quality studies of a positive effect for DMT in reducing depression in adults. Our positive conclusion offers additional and stronger support to existing evidence from previous reviews of DMT for depression (e.g., Meekums et al., 2015) and dance/DMT for symptoms of depression (Koch et al., 2014). Furthermore, we have found that moderate to high quality studies demonstrate strong impact, the strongest possible, when a summary result of the effect size of an intervention is calculated. Additional results, albeit tentative, support the overall conclusion that DMT is a useful intervention in the treatment of depression and offer information about useful trends.

Still, studies that involved children, adolescents and older people were generally missing from this review, confining our positive conclusions to adults with depression only. We were not able to conclude with any confidence whether DMT is beneficial for both men and women. Similarly, in this review, although there were findings relating to secondary outcomes, we did not look at them. Future studies should consider both younger and older populations, men and women and, where available the impact of DMT on secondary outcomes such as anxiety, quality of life, self-esteem and body image next to other clinically relevant outcomes including potentially physiological or neurocognitive changes. Further research attention is also needed on the degree to which positive results, indicated in some of our calculations, can be sustained at a follow up stage and what is the impact of the process of therapy on levels of depression through, for example, mid assessments.

The use of dance, the embodied therapeutic relationship, unearthing and working through difficult issues non-verbally and integrating possible discoveries and solutions creatively may be some reasons for the positive impact of DMT. However, further research on the active ingredients of DMT is needed that can offer further confidence about whether these are indeed helpful factors in the treatment of depression. In-depth study of the manuals of the different types of DMT practice used can result in further refinement of what we think is currently responsible for the significant effects of DMT on reducing depression. A new project called “Arts for the Blues” (Haslam et al., 2019; Karkou et al., 2019; Parsons et al., in press) may offer relevant support in this direction for DMT as well as the other arts therapies.

Furthermore, we propose that studies need to take place where DMT is not simply added to TAU and compared to TAU alone, but it is also compared with other, regularly available, treatment options. Controlling for other forms of psychotherapy such as counseling, art forms such as dance and music, and either group work or recreational activities will also be of considerable benefit as a way of both narrowing down the active ingredients of the intervention and providing comparable results with other, widely used treatments. A new study recently funded by the UK National Health Service might act as a response to this need where DMT, next to art therapy and music therapy, is compared with person-centered counseling (Carr, 2018). This new study, the largest in the field that we know of, will not focus on depression only but on diverse mental health diagnoses to reflect the mixed groups present in regular practice. Still, data extracted from this study for DMT for people with depression will be of particular interest, making a substantial contribution to field.

Finally, the type of approach used, its frequency and overall dosage need to be further explored leading to an associated clinical guideline that takes into account the severity of depression. Such guidelines are currently available for psychological treatment options and exercise (see NICE, 2018), but information about the contribution of DMT is still missing, as are calculations around the cost and cost effectiveness of this intervention. Future developments in this direction are now urgently needed, especially given the positive results of this review.

## Author Contributions

VK was responsible for organizing, drafting, and finalizing the current paper. She also completed the systematic search with BM for the Cochrane review, i.e., the first search, and with SA during the second search. She also guided the statistical analysis for the current review. SA contributed to the systematic review, performed the statistical analyses, and contributed to the writing and editing of the text. BM led the Cochrane review, acted as a referee for the current review and edited the final paper. AZ contributed to revisions and edits of the paper.

### Conflict of Interest Statement

VK and BM are dance movement therapists registered with ADMP UK and as such may be seen as having invested interest in demonstrating the effectiveness of the intervention. SA, originally a speech and language therapist and a dancer, is completing her doctoral studies on DMT. AZ is a psychologist who has been researching arts therapies in her doctoral and post-doctoral work. The submitted work was not carried out in the presence of any other personal, professional, or financial relationships that could potentially be construed as a conflict of interest.

## Footnotes

^1^The discipline in the UK is formally called Dance Movement Psychotherapy to reflect the fact that it is regarded as a form of psychotherapy. It is defined as “…a relational process in which client/s and therapist engage creatively using body movement and dance to assist integration of emotional, cognitive, physical, social and spiritual aspects of self.” (Association of Dance Movement Psychotherapy UK – ADMP UK, 2013, p.1). The discipline is also known under other names: in the USA is known as Dance/Movement Therapy, in Australia as Dance-Movement Therapy, in Germany as Dance Therapy. Other terms used interchangeably are: Dance Psychotherapy, Movement Psychotherapy and Movement Therapy. We will use the term Dance Movement Therapy (DMT) as a term with international recognition to refer to all of these different names for the same profession.

^2^Mirroring: a technique in which the dance movement therapist attempts to find ways of experiencing the feelings of clients by taking on and reflecting back to the client some of their expressive movements.

^3^In Authentic movement, internal sensations are focused upon as they give rise to associated movement.

^4^VK and BM were the reviewers in the first search; VK and SA were the reviewers in the second review and BM acted as a referee.

^5^It was published in Chinese and translated with support from the Cochrane Collaboration by Dr Li Weixiao.
